# Hidden pesticidal diversity within the *Bacillus cereus* group: expanding the concept beyond *Bacillus thuringiensis*

**DOI:** 10.1093/sumbio/qvag029

**Published:** 2026-07-22

**Authors:** Diego Herman Sauka, Leopoldo Palma

**Affiliations:** Instituto Nacional de Tecnología Agropecuaria (INTA), Instituto de Microbiología y Zoología Agrícola (IMYZA), Hurlingham,1686, Argentina; Consejo Nacional de Investigaciones Científicas y Técnicas (CONICET), Ciudad Autónoma de Buenos Aires, Ciudad Autónoma de Buenos Aires,1425, Argentina; Departamento de Genética, Laboratorio de Control Biotecnológico de Plagas, Instituto BIOTECMED, Universitat de València, València,46100, Spain

**Keywords:** *Bacillus thuringiensis*, *Bacillus cereus* group, *Bacillus toyonensis*, pesticidal proteins, accessory genome, microbial biocontrol, horizontal gene transfer, sustainable agriculture

## Abstract

For over a century, *Bacillus thuringiensis* has been regarded as the primary microbial source of pesticidal proteins used in agriculture and vector control. Its defining phenotype, the production of parasporal crystals composed mainly of Cry and Cyt proteins, has shaped both scientific understanding and regulatory frameworks. However, advances in whole genome sequencing reveal that pesticidal traits are not restricted to a single species but are distributed across members of the *Bacillus cereus* group. This distribution is largely driven by the mobility of toxin-associated genetic elements across closely related genomic backgrounds. Here, we propose that this observation reflects a broader, dynamic, and largely unexplored functional diversity. Using the emerging case of *Bacillus toyonensis* biovar Thuringiensis, we argue that the current species centered paradigm may be limiting the discovery of novel pesticidal diversity and propose a shift toward a function centered framework in microbial biocontrol. We suggest that embracing this perspective will not only refine our understanding of microbial evolution but also open new avenues for the development of sustainable, next generation biopesticides. Furthermore, it may facilitate the systematic discovery of previously overlooked pesticidal diversity hidden within historical microbial collections.

**Sustainability Statement**

This perspective article is associated with UN Sustainable Development Goal 12 (Responsible Consumption and Production) as the primary focus. It advances sustainable pest management by expanding microbial biocontrol beyond *Bacillus thuringiensis* to include non-pathogenic and environmentally safe members of the broader *Bacillus cereus* group. Harnessing this functional diversity enables the discovery of novel pesticidal proteins with distinct modes of action, reducing reliance on chemical pesticides and minimizing environmental impacts, while improving the durability of bio-based solutions. Relevant additional SDGs: SDG 3 (Good Health and Well-being), SDG 2 (Zero Hunger), SDG 15 (Life on Land).

## Rethinking a century-old paradigm

The bacterium *B. thuringiensis* represents a key model system for understanding the evolution and diversification of pesticidal traits within the *B. cereus* group, has been a cornerstone of biological pest control for over a century; although historically defined by its entomopathogenic activity, accumulating evidence highlights a broader pesticidal spectrum, including nematicidal effects, associated with its diverse repertoire of pesticidal proteins (Slamti and Lereclus 2026). In parallel, global environmental change is reshaping microbial dynamics and promoting the emergence of novel biological threats across agroecosystems and food systems, highlighting the need to reassess current biocontrol paradigms (Rees and Threlfall 2025).

Since its original description (Berliner 1915), it has been widely applied in agriculture and public health to control diverse insect pests (Slamti and Lereclus 2026). Its success is largely attributed to the production of parasporal crystals containing Cry and Cyt proteins, which display high specificity and toxicity toward susceptible target organisms (Slamti and Lereclus 2026). Additional crystal associated pesticidal proteins have been described, including members of the Tpp and Mpp families, which are also frequently plasmid encoded and incorporated into parasporal crystal inclusions (Best *et al*. 2022, de Oliveira *et al*. 2023). Beyond crystal associated proteins, the pesticidal repertoire of *B. thuringiensis* also includes secreted proteins produced during the stationary growth phase, historically referred to as vegetative insecticidal proteins (Vip), encompassing families such as Vpb1/Vpa2 (formerly Vip1/Vip2), Vip3, and Vpb4 (formerly Vip4) (Berry *et al*. 2025, Slamti and Lereclus 2026).

Historically, this phenotype became synonymous with species identity. The presence of parasporal crystals during sporulation was adopted as the defining diagnostic trait of *B. thuringiensis*, leading to the classification of environmental isolates primarily on this basis (Schnepf *et al*. 1998). This approach reinforced a conceptual model in which insecticidal activity was viewed as an intrinsic property of a single species.

This long-standing paradigm, while historically successful, may now be constraining our ability to fully explore and exploit microbial diversity for biocontrol innovation. Although pesticidal and biocontrol activities have also been reported in *Bacillus* species outside the *B. cereus* group, including *Bacillus subtilis* (Xia *et al*. 2024), the present perspective focuses specifically on the evolutionary dynamics of pesticidal traits within the *B. cereus* group, where extensive sharing of accessory genetic elements creates a unique framework for understanding functional diversification.

However, this paradigm was established in the absence of genomic information and assumes that phenotype reliably reflects phylogeny, an assumption that is increasingly challenged by genome-based analyses (Carroll *et al*. 2020). As a result, the traditional view of *B. thuringiensis* may represent an oversimplified interpretation of a more complex and dynamic biological system.

## Genomes reveal a different reality

The widespread application of whole genome sequencing has transformed bacterial taxonomy and systematics (Riesco and Trujillo 2024). Within this framework, the *B. cereus* group has emerged as a model for understanding the limitations of phenotype-based classification (Carroll *et al*. 2020).

This group encompasses closely related species such as *B. cereus, Bacillus anthracis, B. thuringiensis, Bacillus wiedmannii*, and *Bacillus toyonensis* (Jiménez *et al*. 2013, Miller *et al*. 2016, Ehling-Schulz *et al*. 2019). Despite their distinct ecological roles and phenotypes, these organisms share highly similar chromosomal genomes, often complicating species delineation based on genomic criteria alone (Liu *et al*. 2015, Sauka *et al*. 2026a).

A key insight from genomic studies is that many defining ecological traits are encoded on plasmids rather than chromosomes (Finks and Martiny 2023), often in combination with chromosomal determinants of ecological fitness. This includes virulence factors such as anthrax toxins in *B. anthracis* (Brossier and Mock 2001) and pesticidal Cry, Cyt, and Vip proteins in *B. thuringiensis* (Slamti and Lereclus 2026). Consequently, phenotype and genotype may exhibit context-dependent coupling, challenging traditional taxonomic frameworks (Liu *et al*. 2015). These observations suggest that ecological functions encoded within the accessory genome may provide a complementary perspective for understanding diversification within the *B. cereus* group.

Rather than replacing phenotype-based frameworks, genome-based analyses provide an additional layer of resolution for interpreting diversity within the *B. cereus* group.

## Insecticidal traits as mobile functions

The pesticidal activity of *Bacillus* species is largely determined by genes located on mobile genetic elements (Brossier and Mock 2001, Slamti and Lereclus 2026). Cry and Cyt toxin genes are typically carried on plasmids capable of horizontal transfer between strains (Gonzalez *et al*. 1982, Thomas *et al*. 2001). This mobility enables the dissemination of pesticidal traits across diverse genomic backgrounds within the *B. cereus* group (Hinnekens *et al*. 2022).

From an evolutionary perspective, this suggests that pesticidal activity is better understood as a transferable functional module rather than a species-specific characteristic (Pal *et al*. 2023). In this context, a functional module refers to a set of genetically linked determinants, frequently associated with mobile genetic elements, that collectively confer a specific ecological function such as pesticidal activity. These modules can move across closely related genomic backgrounds through horizontal gene transfer, allowing ecological traits to spread independently of chromosomal lineage. This concept aligns with broader models of microbial evolution in which accessory genomes play a central role in ecological adaptation and functional diversification (Croll and McDonald 2012).

Within this framework, the classical definition of *B. thuringiensis* becomes less a reflection of a distinct lineage and more a representation of a particular functional state. Here, a functional state refers to the phenotypic condition resulting from the acquisition and expression of a particular functional module within a given genomic background. Consequently, the “Thuringiensis” phenotype may emerge across multiple members of the *B. cereus* group that independently acquire and maintain toxin-encoding genetic elements.

## A signal of hidden diversity: *B. toyonensis* biovar Thuringiensis

Recent genomic and phenotypic characterizations have fundamentally challenged the species-specific boundary of pesticidal activity. The “Thuringiensis” phenotype, historically defined by the presence of parasporal crystals, is now understood to be a mobile functional state rather than a fixed phylogenetic marker. The most compelling evidence for this shift is the identification of crystal forming strains genomically assigned to *B. toyonensis* (Sauka *et al*. 2022, 2026a and 2026b). Following the nomenclature proposed by Carroll *et al*. (2020), these strains are now classified as *B. toyonensis* biovar Thuringiensis (NCBI: txid2923195), a designation that acknowledges the chromosomal lineage while highlighting the acquired pesticidal function (Sauka et al. 2022, 2026b).

This phenomenon is further supported by similar findings in other members of the group, such as the mosquitocidal strain FCC41, which was recently reclassified as *B. wiedmannii* biovar *thuringiensis* (Lazarte *et al*. 2018).

Together, these cases suggest that the “Thuringiensis” phenotype may not represent a strictly monophyletic lineage, but rather a functional state that can arise across different genomic backgrounds.

The observations described above are consistent with the concept of pesticidal functions as mobile genetic modules acting within a shared accessory gene pool. The primary driver of this diversity is horizontal gene transfer acting on the accessory genome. While members of the *B. cereus* group share nearly identical core genomes, variation in pesticidal phenotypes reflects differential acquisition of toxin-encoding elements rather than lineage divergence. This explains why strains such as MC28, previously assigned to *B. thuringiensis*, can be reassigned, based on genome-based comparisons, to *B. toyonensis* backgrounds carrying pesticidal gene repertoires active against lepidopteran and dipteran pests (Sauka *et al*. 2026a). Notably, the corresponding NCBI record is flagged with a “taxonomy check failed” warning and a suppressed status (Genome assembly ASM30047v1), indicating that ANI-based comparisons are inconsistent with the submitted species assignment.

The functional plasticity of this biovar is more extensive than previously recognized. Beyond traditional insect targets, recent findings have identified *B. toyonensis* biovar Thuringiensis strain Bto_UNVM-42 as a promising candidate for the biological control of plant parasitic nematodes (Sauka *et al*. 2026b). This expansion into bionematicidal activity may suggest that the “Thuringiensis” phenotype represents a versatile ecological strategy for soil dwelling *Bacillus*.

Rather than representing isolated anomalies, these cases likely reflect a widespread but underrecognized functional diversity within the *B. cereus* group. Historical classification practices, which relied almost exclusively on the presence of parasporal crystals to assign species names, have inadvertently masked the true evolutionary origin of many biocontrol strains. Today, advances in whole genome sequencing and bioinformatics are correcting these legacy misidentifications in databases like GenBank, enabling a more accurate mapping of the *Bacillus* functional landscape (Schoch *et al*. 2020). This raises a critical and forward-looking question: how much pesticidal functional diversity remains hidden within what is currently labeled as *B. thuringiensis*. Reevaluating these collections is not just a taxonomic necessity; it is a strategic imperative to discover novel pesticidal proteins and develop the next generation of sustainable, multi target biocontrol solutions.

## The overlooked potential of microbial collections

Microbial culture collections constitute extensive repositories of biodiversity accumulated over decades of research (Lima 2025). However, within these collections, many *B. thuringiensis* strains were historically classified mainly using phenotype-based approaches, often relying on traits such as the presence of parasporal crystals to infer taxonomic identity.

Reevaluating these collections using genomic tools may provide an opportunity to uncover previously unrecognized diversity. This effort may be particularly valuable for identifying strains carrying novel combinations of genomic backgrounds and pesticidal determinants that remained obscured under historical phenotype-based classifications. It is plausible that a proportion of strains currently assigned to *B. thuringiensis* may belong to other genomic species within the *B. cereus* group, reflecting historical misclassification driven by function-based rather than phylogeny-based criteria (Sauka *et al*. 2026b).

Such reanalysis could reveal novel combinations of genomic backgrounds and pesticidal traits, consistent with the concept of pesticidal functions as mobile genetic modules, expanding the repertoire of bioactive compounds available for pest control applications (Sauka *et al*. 2026b).

## Implications for sustainable microbiology

The recognition of a broader diversity of pesticidal *Bacillus* has important implications for sustainable microbiology. Expanding the search beyond *B. thuringiensis* increases the likelihood of identifying novel pesticidal proteins with distinct modes of action (Lazarte *et al*. 2018, Sauka *et al*. 2022 , 2026a).

This diversification is particularly relevant in the context of resistance management, especially in crops expressing *B. thuringiensis* pesticidal proteins. The widespread use of a limited number of pesticidal proteins has contributed to the emergence of resistant insect populations (Huang 2021). A more diverse pool of bioactive compounds may help mitigate this risk and improve long-term efficacy.

In addition, integrating genomic and functional data enables more predictive approaches to strain selection and development (Sauka *et al*. 2023, 2026a). Such approaches may accelerate the discovery of novel pesticidal proteins and improve the rational design of next-generation microbial biocontrol products. This shift moves the field away from purely empirical screening toward a more rational, data-driven discovery process, aligning with emerging strategies for designing next-generation microbial bioinputs based on mechanistic understanding (Sauka *et al*. 2026c).

## Toward a function-centered framework

The observations presented here support a transition from a species-centered to a function-centered framework in microbial biotechnology. Rather than viewing pesticidal activity as an intrinsic property of a specific species, this perspective considers ecological functions as dynamic traits that can be acquired, maintained, and redistributed across related genomic backgrounds. In this view, ecological traits such as pesticidal activity can be viewed as components of a shared functional repertoire rather than properties of individual species (Sauka *et al*. 2026b, Lazarte *et al*. 2018).

This perspective facilitates a more flexible and accurate understanding of microbial diversity, particularly in systems shaped by horizontal gene transfer and accessory genomes. It also enables the identification of functionally relevant traits across a broader range of genomic backgrounds.

In practical terms, this shift reframes microbial biocontrol from a taxonomy-driven discipline to a function-driven discovery pipeline, where the primary unit of interest is the trait rather than the organism that carries it.

## Concluding perspective

The recognition of *B. toyonensis* biovar Thuringiensis, together with similar findings in *B. wiedmannii* biovar *thuringiensis*, challenges the long-standing assumption that pesticidal activity is exclusive to *B. thuringiensis* within the *B. cereus* group. Instead, it reveals a dynamic evolutionary landscape in which functional traits are mobile and widely distributed across this group.

These observations suggest that our current view of pesticidal diversity within the *B. cereus* group remains incomplete. The growing availability of genomic data and analytical tools provides new opportunities to reexamine historical strain collections and public databases, potentially uncovering additional lineages carrying previously overlooked pesticidal functions. Such efforts may not only improve our understanding of the evolution of these traits but also expand the pool of biological resources available for pest management.

Future research should prioritize the integration of comparative genomics, functional characterization, and ecological studies to identify novel pesticidal determinants and evaluate their practical relevance. Attention should be given to strains that fall outside traditional taxonomic expectations yet harbor traits of interest for biocontrol.

From a sustainability perspective, broadening the search for pesticidal functions beyond historically recognized species may facilitate the discovery of new biological control agents and modes of action, helping to diversify pest management strategies and reduce dependence on conventional chemical pesticides. In this sense, recognizing and exploring hidden functional diversity is not only a question of taxonomy or evolution, but also an opportunity to strengthen the contribution of microbial biotechnology to more sustainable agricultural systems.

## Data Availability

This article does not report new data.
